# REAP: A two minute cell fractionation method

**DOI:** 10.1186/1756-0500-3-294

**Published:** 2010-11-10

**Authors:** Keiko Suzuki, Pinaki Bose, Rebecca YY Leong-Quong, Donald J Fujita, Karl Riabowol

**Affiliations:** 1 Department of Biochemistry & Molecular Biology, Faculty of Medicine, University of Calgary, 3330 Hospital Drive NW, Calgary, T2N 4N1, Canada; 2 Department of Oncology, Faculty of Medicine, University of Calgary, 3330 Hospital Drive NW, Calgary, T2N 4N1, Canada

## Abstract

**Background:**

The translocation or shuttling of proteins between the nucleus and cytoplasm (nucleocytoplasmic transport [NCPT]) is often a rapid event following stimulation with growth factors or in response to stress or other experimental manipulations. Commonly used methods to separate nuclei from cytoplasm employ lengthy steps such as density gradient centrifugation which exposes cells to non-physiological hyperosmotic conditions for extended time periods resulting in varying degrees of leakage between the nucleus and cytoplasm. To help maintain and quantify nuclear:cytoplasmic ratios of proteins, agents such as leptomycin B have been employed to be able to better analyze NCPT by inhibiting nuclear export. To track NCPT in the absence of these experimental manipulations that could introduce unknown artefacts, we have developed a rapid method that appears to produce pure nuclear and cytoplasmic fractions, suitable for obtaining accurate estimates of the nuclear:cytoplasmic ratios of proteins known to undergo NCPT.

**Findings:**

We have developed a **R**apid, **E**fficient **A**nd **P**ractical (**REAP**) method for subcellular fractionation of primary and transformed human cells in culture. The REAP method is a two minute non-ionic detergent-based purification technique requiring only a table top centrifuge, micro-pipette and micro-centrifuge tubes. This inexpensive method has proven to efficiently separate nuclear from cytoplasmic proteins as estimated by no detectible cross-contamination of the nucleoporin and lamin A nuclear markers or the pyruvate kinase and tubulin cytoplasmic markers. REAP fractions also mirrored TNFα induced NF-κB NCPT observed in parallel by indirect immunofluorescence.

**Conclusions:**

This method drastically reduces the time needed for subcellular fractionation, eliminates detectable protein degradation and maintains protein interactions. The simplicity, brevity and efficiency of this procedure allows for tracking ephemeral changes in subcellular relocalization of proteins while maintaining protein integrity and protein complex interactions.

## Findings

Subcellular fractionation was first described by Albert Claude in 1946 [1,2]. He wrote: "The physiology of the cell cannot be fully understood unless we succeed in determining the constitution of its parts,..." [2]. Subsequently, Claude's method was improved upon by Hogeboom, Schnieder and Palade to obtain the nuclear fraction which was discarded in Claude's original method along with cell debris [3]. Christian de Duve pioneered the use of sucrose density gradients to fractionate cells in 1951 [4,5] and subsequent researchers have developed various additional modifications [6-8]. Over the last 60-70 years, cell fractionation has provided biologists with valuable reagents to provide insight into cellular architecture, composition and function of cellular organelles. The nucleus and the cytoplasm have very distinct macromolecular composition and separation of nuclear and cytosolic fractions is proving very useful for proteomic analysis [9]. A majority of the established methods of subcellular fractionation are based on subtle variations of the sucrose density gradient method, often with addition of detergents to solubilize membrane proteins [10,11]. However, most of these methods are time consuming and may not be necessary when examining protein localization and complex formation in the nucleus and cytoplasm in cultured cells. Here we introduce a **R**apid **E**fficient **A**nd **P**ractical (**REAP**) nuclear/cytoplasmic separation protocol using various cultured cells as the starting material. The results obtained from this procedure have been validated by western blotting with two different nuclear and cytoplasmic markers in four different cell types including primary human diploid fibroblasts (HDF) and have also been used in immunoprecipitation-western analyses with good results. The REAP method also performed well for TNFα induced NF-κB NCPT, corroborating changes in subcellular localization visualized in parallel by indirect immunofluorescence in mouse embryonic fibroblast cells.

## Methods

### REAP method

All cells used in this study were obtained from the American Type Culture Collection (ATCC). HeLa (human cervical cancer, ATCC# CCL-13), HCT116 (human colorectal cancer, ATCC# CCL-247), HEK293 (adenovirus infected human embryonic kidney, ATCC# CRL-1573) and HS68 (normal HDF, ATCC# CRL-1635) cells grown as monolayers in 10 cm diameter dishes were washed in ice-cold phosphate buffer saline (PBS) pH 7.4, scraped from culture dishes on ice using a plastic cell scraper and collected in 1.5 ml micro-centrifuge tubes in 1 mL of ice-cold PBS. After centrifugation (a "pop-spin" for 10 sec in an Eppendorf table top microfuge), supernatants were removed from each sample and cell pellets were resuspended in 900 μL of ice-cold 0.1% NP40 (Calbiochem, CA, USA) in PBS and triturated 5 times using a p1000 micropipette (Gilson, WI, USA). 300 μL of the lysate was removed as "whole cell lysate" and 100 μL of 4 × Laemmli sample buffer was added to it, then kept on ice until the sonication step. The remaining (600 μL) material was centrifuged for 10 sec in 1.5 ml micro-centrifuge tubes and 300 μl of the supernatant was removed as the "cytosolic fraction". 100 μL of 4 × Laemmli sample buffer was added to this fraction and boiled for 1 min. After the remaining supernatant was removed, the pellet was resuspended in 1 ml of ice-cold 0.1% NP40 in PBS and centrifuged as above for 10 sec and the supernatant was discarded. The pellet (~20 μL) was resuspended with 180 μL of 1 × Laemmli sample buffer and designated as "nuclear fraction". Nuclear fractions and whole cell lysates that contained DNA were sonicated using microprobes (Misonix, NY, USA) at level 2, twice for 5 sec each, followed by boiling for 1 min. 10 μL, 10 μL and 5 μL of whole cell lysate, cytoplasmic and nuclear fractions, respectively, were loaded and electrophoresed using sodium dodecyl sulfate polyacrylamide gel electrophoresis (SDS-PAGE) [12] and transferred to nitrocellulose membranes (Pall Life Sciences, FL, USA). Membranes were incubated with anti-pyruvate kinase (Santa Cruz, CA, USA) or anti-α-tubulin (Calbiochem, CA, USA) antibodies as cytoplasmic markers or anti-lamin A (Santa Cruz, CA, USA) or anti-nucleoporin (Santa Cruz, CA, USA) as nuclear markers after blocking with 3% bovine serum albumin in 0.1% tween 20-PBS (t-PBS). Membranes were washed with t-PBS followed by incubation with HRP-conjugated anti-rabbit or anti-mouse secondary antibody. After washing with t-PBS, target protein signals were detected by ECL (GE Healthcare, Buckinghamshire, UK) on Kodak X-ray film.

### TNFα treatment and NF-κB NCPT

Wild-type MEFs (mouse embryonic fibroblast, ATCC# CRL-2991) were grown in 3 cm dishes with or without glass cover slips. After 1 ng/ml TNFα treatment for 15 min [13,14], cells were harvested by the REAP method as described above. Anti NF-κB p65 (Santa Cruz, CA, USA), anti -α-tubulin (Calbiochem, CA, USA) and anti-hnRNP C1/C2 (Santa Cruz, CA, USA) antibodies were used for western-blotting analysis. For immunofluorescence, cells grown on cover slips were fixed with methanol and processed as previously described [15]. Briefly, fixed cells were incubated with rabbit anti NF-κB p65 (Santa Cruz, CA, USA) after blocking with 3% BSA, then washed with t-PBS, followed by Alexa-conjugated anti-Rabbit IgG (Invitrogen, Oregon, USA) incubation. After washing with t-PBS, nuclei were stained by 4',6'-Diamidino-2-phenylindole (DAPI) to visualize DNA.

## Results

**Figure 1:**
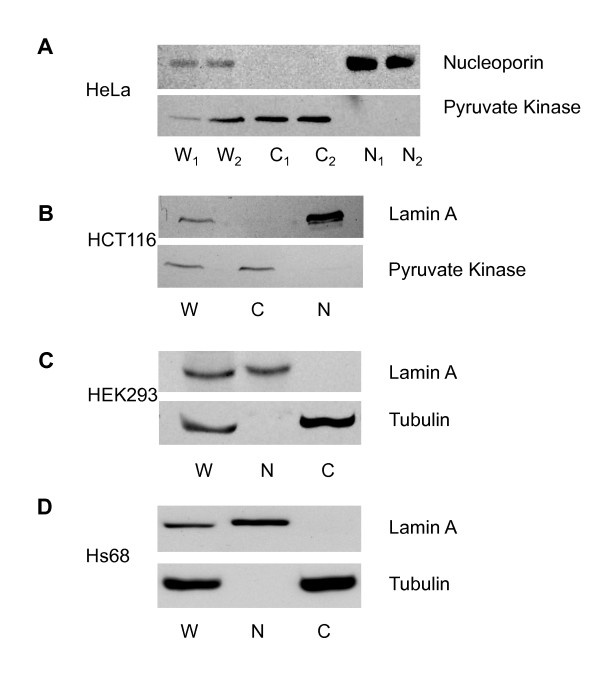
**Separation of nuclear and cytoplasmic proteins by differential centrifugation in non-ionic detergent**. Panel A, B, C and D show fractionation results from HeLa, HCT116, HEK293 and Hs68 cells, respectively. The upper panel in each section shows immunoblotting results for nuclear markers (nucleoporin or lamin A) and the lower panels show the same for cytoplasmic markers (pyruvate kinase or tubulin). Subcellular fractions are abbreviated as W for whole cell lysate, C for cytoplasmic fraction and N for nuclear fraction. In panel A, HeLa cells were obtained from two independent culture dishes, and results are shown as W_1_, W_2_, C_1_, C_2_, N_1 _and N_2_.

Immunoblotting results from HeLa, HCT116, HEK293 and HS68 cells are shown in Figure 1, panels A, B, C and D, respectively. Bands corresponding to all the marker proteins were observed in whole cell lysates in each of the four cell types with no cross contamination between nuclear and cytoplasmic fractions and negligible protein degradation. For example, the nuclear markers nucleoporin in Hela cells and lamin A in HCT116, HEK293 and HS68 cells, were not detected in cytoplasmic fractions. Conversely, the cytoplasmic markers (pyruvate kinase and tubulin) were not detected in nuclear fractions.

**Figure 2:**
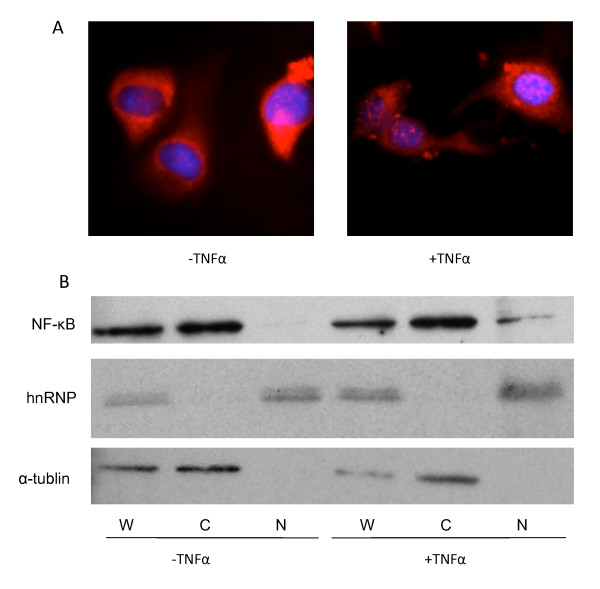
**Visualizing TNFα-induced NF-kappaB translocation**. MEF cells were treated with 10 ng/ml of TNFα for 15 min and processed by the REAP method for western blotting, or were fixed for indirect immunofluorescence, staining for NF-kB (red) and DNA (blue). Panel A shows that staining for NF-kB was primarily cytoplasmic in the absence of stimulation (left panel) and that a significant amount of NF-kB staining appeared in the nucleus after TNFα treatment (right panel). Panel B shows whole cell (W), cytoplasmic (C) and nuclear (N) fractions prepared by REAP and blotted for NFkB, hnRNP (nuclear marker) and α-tublin (cytoplasmic marker).

We next wished to test whether the REAP method would faithfully reflect subcellular localization and alterations in subcellular localization looking at a protein known to partition between the nucleus and cytoplasm. TNFα-induced NF-κB translocalization was tracked in parallel using the REAP method followed by western blotting and compared to indirect immunofluorescence. NF-κB was primarily cytoplasmic in unstimulated cells (Figure 2A, left panel), but significant amounts were observed to translocate from the cytoplasm to nucleus after TNFα stimulation (Figure 2A, right panel). Blotting of REAP fractions (Figure 2B) showed that nuclear (snRNP staining) and cytoplasmic (α-tubulin staining) fractions showed little if any cross-contamination consistent with results in Figure 1. Furthermore, a significant amount of NF-κB was seen in the nucleus after, but not before TNFα stimulation, consistent with immunofluorescence results.

Processing times and required reagents for this REAP method are compared with a standard sucrose density gradient procedure taken from the Laboratory Handbook-Cell Biology [16] in Figure 2. Since homogenization and sucrose gradient layering steps are not required in the REAP protocol and centrifugation times are significantly shorter in the method, we have decreased the handling time to approximately 2 versus 20 minutes compared to this standard sucrose density gradient method. This decrease in processing time significantly reduces protein degradation, enhancing the probability of detection of proteins with short half-lives or marginal solubility and helps maintain protein complexes in nuclear and cytoplasmic samples. Low concentrations of non-ionic detergent (0.1%) disrupt cytoplasmic, but not nuclear membranes, and short centrifugation times allow intact nuclei to be pelleted leaving soluble cytosolic proteins in the supernatant. Increasing the detergent concentration to 0.5% leads to the contamination of cytoplasmic samples with nuclei (data not shown), due to the permeabilization of nuclei at higher detergent levels. Modified versions of this method using combinations of non-ionic detergents such as 0.05% NP40 and 0.05% Tween 20 have been successfully used for examination of protein-protein interactions by co-immunoprecipitation-western analyses for both nuclear [17] and cytoplasmic [15] proteins indicating that solubility is also maintained. These reports support the idea that low detergent concentrations combined with modest mechanical shear forces generated by trituration are effective for very rapid nuclear-cytoplasmic fractionation which maintain protein and protein complex integrity.

## Conclusions

**Figure 3:**
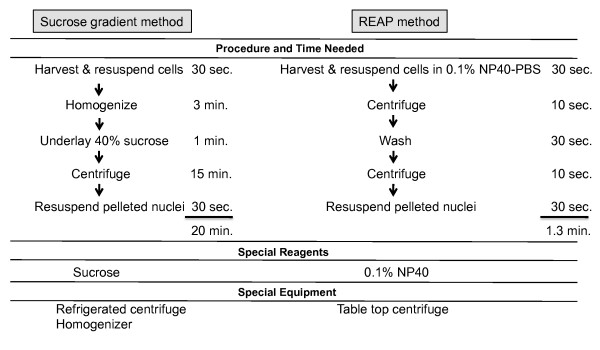
**Comparative flowchart of sucrose gradient method and the non-ionic detergent method**. The procedures are compared regarding the time, reagents and equipment required for both methods.

We have developed and optimized a rapid and simple method for preparing nuclear and cytoplasmic fractions from cultured normal and transformed cells that requires no specialized equipment (see itemized Protocol in Figure 3). This procedure maintains nuclear and cytoplasmic localization, protein integrity, integrity of protein complexes and solubility, indicating that it should be applicable to many experimental questions. Reagents required and a step-by-step outline are provided in Additional File 1. The REAP method provides clear advantages, particularly for the analysis of protein subcellular relocalization and protein complex interactions.

## List of Abbreviations

Abbreviations used include: ATCC: American Type Culture Collection; ECL: Enhanced ChemiLuminesence; HDF: Human Diploid Fibroblasts; HRP: HorseRadish Peroxidase; NCPT: NucleoCytoPlasmic Transport; NF-κB: Nuclear Factor kappa B; PBS: Phosphate-Buffered Saline; REAP: a Rapid, Efficient And Practical method for subcellular fractionation; SDS-PAGE: Sodium Dodecyl Sulfate PolyAcrylamide Gel Electrophoresis.

## Declaration of Competing interests

The authors declare that they have no competing interests.

## Authors' contributions

KS, PB and RYYLQ generated data shown in the panels of Figure 1, KS and PB wrote the first draft of the manuscript, KS produced Figures 2 and 3 and DF and KR helped to conceive and design experiments and write the manuscript. All authors read and approved the final manuscript.

## Supplementary Material

Additional file 1 **Protocol for REAP nuclear/cytoplasmic fractionation**. The reagents, equipment needed, procedure and solution recipes are outlined in a printable format suitable for lab use. Click here for file
