# Supplementary material for: REAP: A two minute cell fractionation method
Source: BMC Res Notes. 2010 Nov 10;3:294. doi: 10.1186/1756-0500-3-294 (PMC2993727; doi:10.1186/1756-0500-3-294)
Supplement: Additional file 1 — Protocol for REAP nuclear/cytoplasmic fractionation. The reagents, equipment needed, procedure and solution recipes are outlined in a printable format suitable for lab use. [file 1756-0500-3-294-S1.PDF]

Protocol for REAP nuclear/cytoplasmic fractionation

REAGENTS
NP40 (Calbiochem, San Diego, CA, USA)

EQUIPMENT
Table-top centrifuge (Eppendorf, Barkhausenweg, Hamburg, Germany)
P1000 micropipette (Gilson, Middleton, WI, USA)
Micropipet tip for p1000 micropipet (Axygen, Union City, CA, USA)
1.5ml microcentriguge tube (Axygen, Union City, CA, USA)
Cell scraper (Corning, Middleton, WI, USA)
Sonicator with microprobes (Misonix, Farmingdale NY, USA)

PROCEDURE

[Chill all reagents on ice.

Cell collection

1. Remove all culture medium from cell culture dish
2. Wash cell surface twice with ice-cold PBS
3. Add ice-cold PBS into cell culture dish (1 ml per 10 cm diameter dish)
4. Scrape cells with cell scraper
5. Collect cells with PBS into 1.5 ml microcentrifuge tube
6. Pop-Spin the tube for 10 sec. with table top centrifuge
7. Discard supernatant

[Keep cell dishes and tubes on ice

Fractionation: 0.1% NP40-PBS treatment

8. Triturate (pipet up & down) cell pellet 5 times with ice-cold 0.1% NP40-PBS (900-1000 µL for cells from 10 cm diameter dish) using p1000 micropipette cut about 3 mm off at the end to enlarge the opening.

[Remove an aliquot into a fresh tube as the whole cell sample and keep on ice

9. Pop-Spin the remainder in the tube for 10 sec. with table top centrifuge
10. Transfer the supernatant to new tube and keep it on ice

[This is the cytoplasmic fraction.

11. Resuspend the pellet with ice-cold 0.1% NP40-PBS (1 ml for cells from 10cm diameter dish, triturate once)
12. Pop-Spin the tube for 10 sec. with table top centrifuge
13. Discard supernatant

[This is the nuclear pellet which should be white compared to the yellowish tone of the washed whole cell pellet at step 7.

Sample preparation for SDS-PAGE

- Whole cell lysate (from step 8)
  14. Mix 3 vol. of samples and 1 vol. of 4x Laemmli sample buffer
  15. Sonicate using microprobes at level 2, twice for 5 sec each on ice
  16. Boil for 1-4 min.
- Cytoplasmic fraction (from step 10)
  14. Mix 3 vol. of samples and 1 vol. of 4x Laemmli sample buffer
  15. Boil for 1-4 min.
- Nuclear fraction (from step 13)
  14. Resuspend the pellet with 1x Laemmli sample buffer (~200 µL for pellet from 10cm diameter dish)
  15. Sonicate using microprobes at level 2, twice for 5 sec. each on ice
  16. Boil for 1-4 min.

Sample preparation for immunoprecipitation

- Whole cell lysate (from step 8)
  14. Sonicate using microprobes at level 2, twice for 5 sec each on ice
  15. Centrifuge at 8000 x g for 30 sec. at 4 °C, transfer supernatant to a fresh tube
  16. The supernatant can be immunoprecipitated directly, with or without addition of protease inhibitors and/or an equal volume of 2x RIPA buffer
- Cytoplasmic fraction (from step 10)
  14. Centrifuge at 8000 x g for 30 sec. at 4 °C, transfer to a fresh tube
  15. The supernatant can be immunoprecipitated directly, with or without addition of protease inhibitors and/or an equal volume of 2x RIPA buffer
- Nuclear fraction (from step 13)
  14. Resuspend the pellet with ice-cold 0.1%NP40-PBS (~500 µL for pellet from 10 cm diameter dish)
  15. Sonicate using microprobes at level 2, twice for 5 sec each on ice
  16. Centrifuge at 8000 x g for 30 sec. at 4 °C, transfer to a fresh tube
  17. The supernatant can be immunoprecipitated directly, with or without addition of protease inhibitors and/or an equal volume of 2x RIPA buffer

RECIPES

<u>10x PBS (pH7.4)</u> (total volume: 1L)

Ingredient	Volume (g)	Final concentration (mM)
NaCl	80	1370
KCl	2	27

$Na_2HPO_4 \cdot 12H_2O$	29	80
KH_2PO_4	2	15

<u>0.1% NP40-PBS</u> (total volume: 100mL)
10mL of 10x PBS
1mL of 10% (Vol. / Vol.) NP40
make vol. up to 100ml

<u>4x Laemmli sample buffer</u> (total volume: 10mL)

Ingredient	Volume	Final concentration
0.25M Tris-HCl (pH6.8)	10 ml	0.25%
β Mercaptoethanol	2 ml	20%
SDS	0.8 g	8%
Glycerol	2 ml	20%
Bromophenol Blue	0.8 mg	0.008%

2x IP (immunoprecipitation) buffer (total volume: 1L)

Ingredient	Volume	Final concentration (mM)
0.5 M Tris-HCl (pH 7.4)	20 ml	10
1 M NaCl	150 ml	150
1 M KCl	10 ml	10
1 M EDTA	1 ml	1
10% (V/V) NP-40	10 ml	0.1%
1 mg/ml pepstatin	1 ml	1 mg/ml
1 mg/ml aprotinin	1 ml	1 mg/ml
1 mg/ml leupeptin	1 ml	1 mg/ml
